# Direct Observation of Amyloid *β* Behavior at Phospholipid Membrane Constructed on Gold Nanoparticles

**DOI:** 10.1155/2018/2571808

**Published:** 2018-12-02

**Authors:** Keishi Suga, Ying-Chen Lai, Miftah Faried, Hiroshi Umakoshi

**Affiliations:** Division of Chemical Engineering, Graduate School of Engineering Science, Osaka University, 1-3 Machikaneyama-cho, Toyonaka, Osaka 560-8531, Japan

## Abstract

Amyloid *β* (A*β*) is a potential biomarker of Alzheimer's disease (AD), and its fibrillation behavior is of interest and value. In this study, the A*β* behaviors on phospholipid membranes were observed by Membrane Surface-Enhanced Raman Spectroscopy (MSERS) method. Phospholipid (PL) membranes, consisting of DMPC and DMPS with a molar ratio of 9:1, were fabricated on gold nanoparticles with diameter of 100 nm (Au@PL). Enhancement of the Raman intensity of Au@PL was increased by A*β*, with enhancement factor about 40. The H-bonding network was disturbed in presence of NaCl which covered Au@PL and made Au@PL away from one another. When A*β* was applied with Au@PL, the H-bonding network was disturbed just after mixing. As the reaction reaches to equilibrium, A*β* attracted neighbouring Au@PL and induced aggregation of Au@PL which blocked the aggregation prone site of A*β* to inhibit further fibrillation. Based on our method, the A*β* behaviors at lipid membrane surface can be directly observed via enhanced Raman signals.

## 1. Introduction

Amyloid *β* (A*β*) proteins are considered as possible biomarkers in Alzheimer's disease (AD). Fibril plaques, mainly composed of A*β* molecules in *β* sheet structure, are accumulated in the brain tissue of the patients at the final stage of AD, thus to clarify the fibrillation behavior of A*β* is one of the important topics in medical research. Amyloid fibril formation is one of the hallmarks for many neurodegenerative diseases such as Alzheimer's, Parkinson's, and Huntington's diseases. Soluble A*β* is released during normal metabolism but the mechanism underlying the toxic effects of A*β* is unclear. A key component in the pathogenesis of Alzheimer's is the accumulation of the A*β* peptide with 40 and 42 amino acid residues, which could spontaneously self-assemble to form toxic amyloid plaques. Previous studies have supported that the oligomeric and prefibrillar states are more toxic than mature amyloid fibrils [1]. Although the biological mechanisms are not well understood, amyloid beta fibrillation is known to be involved with the progression of Alzheimer's disease [2, 3].

It is reported that A*β* tends to bind to hydrophobic and charged surfaces [4–6]. In addition, the A*β* binding to the surface can lead both to accelerate and to inhibit their fibrillation. In the case of nanoparticle (NP) surface, the size, shape, and surface charge density can be the factors to regulate the A*β* fibrillation [7]. Metal NPs such as gold (AuNP) and silver are applied to analysis and sensing, because of their unique plasmonic properties. Thus, the monitoring of A*β* behaviors by NPs is worth to be investigated. Mirsadeghi et al. reported the effect of AuNP size and shape on the A*β* fibrillation process, in the presence and absence of protein such as fetal bovine serum (FBS) and human plasma (HP) [4]. The fibrillation of A*β* is not involved all sequence from A*β* protein. The aggregation prone sites are known as amino acid sequences of 17-24, 30-36, and 38-42 [8]. The A*β* segment of 17-24 (amino acid sequence: KLVFFAED) includes one positively charged amino acids, while the net charge of A*β* molecule is negative at physiological pH. Kim et al. reported the effect of AuNP size, shape, and surface charge on the fibrillation process of A*β* at brain lipid membranes [6]. For AuNP with different size, the fibrillation became faster as the size getting larger [7]. Cabaleiro-Lago et al. studied the inhibition of A*β* fibrillation by hydrophobic nanoparticles made by polystyrene [5]. In addition, the fibrillation process of A*β* is started from nucleation of monomers to form oligomers and finally becomes fibrils. This process is divided into two phase, one is the lag phase and the other is the elongation phase [9]. It is assumed that AuNP influenced the nucleation step because its inhibition effect was only observed for AuNP added at the early stage of the fibrillation.

To sum up, AuNP inhibits the fibrillation of A*β* via blocking the aggregation prone sites of A*β* monomers. The affinity of A*β* self-aggregation has turned into the binding tendency toward AuNP in the presence of hydrophobic or positively charged AuNP. However, with limited capacity of AuNP to block A*β*, the saturated AuNP may play a role as a nucleus to trigger the fibrillation. By utilizing the advantage of AuNP, surface-enhanced Raman scattering (SERS) technique can be applied to directly monitor the A*β* behaviors at the NP surface. Raman analysis enables us to observe molecules without any reporter (or label), while the Raman intensity is not always strong enough. In a recent work, the NP conjugating with thioflavin T (ThT) has been reported, which enables ultrasensitive detection of amyloid protein [10]. Raman or SERS analyses have advantages because they do not require any probes. However, considering the fact that the A*β* fibrillation can be induced at the lipid membrane surface [1, 8], it is more rational to analyze the A*β* fibrillation behaviors at the lipid membrane surface, without any probing molecules.

The aim of this study is to observe A*β* by Raman spectroscopy. To improve Raman intensity, we focused on Membrane Surface-Enhanced Raman Spectroscopy (MSERS) method, which has developed to investigate the dynamic behaviors of phospholipid membranes [11]. To imitate the biological plasma membrane, the phospholipid mixture composed of 1,2-dimylistoyl-*sn*-glycero-3-phosphocholine (DMPC) and 1,2-dimyristoyl-*sn*-glycero-3-phospho-L-serine (DMPS) with molar ratio of 9 to1 was employed. Then, the A*β* fibrillation behaviors were minored based on Raman, and the fluorescence of ThT.

## 2. Materials and Methods

### 2.1. Materials

DMPC and DMPS were purchased from Avanti Polar Lipids, Inc. (Alabaster, AL, USA). Human A*β* (1-40) was purchased from Peptide Institute (Osaka, Japan). A solution of 100 nm citrate-stabilized Au nanoparticles (3.8 ×10^9^ particles per milliliter) was purchased from Sigma-Aldrich (St. Louis, MO, USA). Other chemicals were purchased from Wako Pure Chemical Industry Ltd. (Osaka, Japan) and were used without further purification.

### 2.2. Preparation of Multilamellar Vesicles (MLVs)

A methanol/chloroform (v/v = 1/2) solution of DMPC/DMPS (molar ratio 9:1) was dried in a round-bottom flask by rotary evaporation under negative pressure at 60°C. The obtained lipid thin film was redissolved in methanol/chloroform solution and the solvent was evaporated again. The lipid thin film was kept under a high vacuum for a minimum of 3 hours and was then hydrated with distilled water to a final concentration of 22 mM at room temperature. The small vesicle was thawed at 60°C and frozen at -80°C to convert into larger multilamellar vesicles (MLVs) [12]. The freezing-thawing treatment was conducted five times. The final solution was kept in refrigerator at 4°C.

### 2.3. Preparation of Phospholipids-Modified Gold Nanoparticles (Au@PL)

The Au@PL was prepared on the basis of a previous report [11]. 100 nm AuNPs were coated with octanethiol by mixing 2 mL of AuNP solution, 1 mL of water, 3 mL of ethanol, 3 mL of chloroform, and 10 *μ*L of octanethiol and stirring for 3 hours at room temperature. The solution was left for 30 minutes until separating into two phases. The bottom phase containing octanethiol-modified AuNPs was extracted into the flask, and the solvent was evaporated under vacuum condition for 3 hours. A methanol/chloroform (v/v = 1/2) solution of DMPC/DMPS (molar ratio 9:1) was added to the flask. The solvent was removed by rotary evaporation at 60°C and then kept under a high vacuum for at least 3 hours. After completely removing the solvent, freezing-thawing treatment was performed five times and crude Au@PL was obtained.

The crude Au@PL suspension was centrifuged at 5000 rpm for 5 minutes to isolate Au@PL from the mixture of Au@PLs and PL vesicles. The precipitated Au@PLs were dispersed in distilled water and the final concentration of PC was 0.45 mM measured with an assay kit (Phospholipid C-Test; Wako Pure Chemical) [13]. The UV-vis spectra of Au@PLs were measured from 400 to 700 nm by UV-vis spectroscopy (UV-1800, Shimadzu, Kyoto, Japan).

### 2.4. Au@PL with Addition of NaCl and A*β* (1-40)

NaCl with final concentration of 0.01, 0.05, and 0.1 M was mixed with purified Au@PL solution within 5 minutes and then detected with UV-vis spectroscopy in 10 mm-path length of quartz cell and Raman spectroscopy by droplet on aluminum foil. A*β* with final concentration of 25 *μ*M was incubated with Au@PL in 37°C water bath for 2 days. Both the initial and 2 days incubation of A*β* were measured by Raman spectroscopy. In order to simplify the experiment, a control group, replacing NaCl/A*β* with distilled water, was measured in the same condition.

### 2.5. Fibrillation Experiment by Thioflavin T (ThT)

A 200 *μ*M of A*β* stock solution was prepared by dissolving 0.55 mg of A*β* protein in 0.1% ammonia solution. A*β* fibrillation was studied in the absence and presence of MLVs/Au@PLs by using ThT assay under Tris-buffer solution (pH=7.5, with 100 mM NaCl) at 37°C water bath. The final concentration for A*β*, PLs, and ThT were 5, 150, and 10 *μ*M, respectively. Then, the change of ThT fluorescence with time was measured from 460 to 510 nm by fluorescence spectroscopy (FP-6500; JASCO, Tokyo, Japan) with the excitation wavelength at 444 nm. The time-dependent fluorescence spectra were fitted by sigmoidal formula as follows:

(1) $$\begin{array}{c} I=I_{min}+\frac{I_{max}-I_{min}}{1+\exp\left[-\left(\left(t-t_{1/2}\right)/\tau \right)\right]}, \end{array}$$

wherein *I*_min_ and *I*_max_ show initial and final fluorescence intensity of ThT at 484 nm, respectively. *τ* represents a constant and t_1/2_ represents the time it takes to get to 50% of I_max_. From the fitted parameters, the time for A*β* to change its conformation for further aggregation (t_lag_) could be calculated as follows:

(2) $$\begin{array}{c} t_{lag}=t_{1/2}-2\tau . \end{array}$$

The value of t_1/2_-t_lag_ indicates the time for the fibril maturation.

### 2.6. Raman Measurement and Analysis

Raman spectra of MLVs, Au@PL and Au@PL with addition of NaCl and A*β* were measured using a confocal Raman microscopy (LabRAM HR-800, Horiba, Ltd., Kyoto, Japan). The 532 nm YAG laser of a 100 mW was used as excitation source and a 20x objective lens was used to focus the laser beam on the 10 *μ*L droplet of sample. Each Raman spectrum was measured with accumulation of 10 s, and averaged by 3 times. The background signal of solvent was subtracted, and the baseline was corrected by multipoint selection. Raman intensity at 2880 and 2850 cm^−1^ are originated from hydrocarbon chains [14, 15]. From Raman spectra of Au@PL, enhancement factor (EF) of each peak was evaluated as follows:

(3) $$\begin{array}{c} EF=\frac{I_{MSERS}/C_{MSERS}}{I_{Raman}/C_{Raman}}, \end{array}$$

wherein *I*_MSERS_ and *C*_MSERS_ represent the Raman intensity and concentration of Au@PL, and *I*_Raman_ and *C*_Raman_ are those of MLVs, respectively.

## 3. Results and Discussion

### 3.1. Characterization of Au@PL

**Figure 1:**
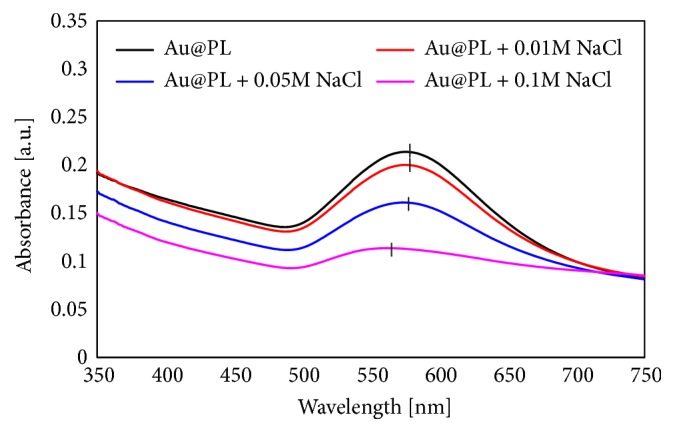
UV-vis spectra and of Au@PL with addition of NaCl in different concentration.

The prepared Au@PL was characterized using UV-vis spectroscopy (Figure 1). The localized surface plasmon resonance (LSPR) bands of untreated AuNPs and Au@PL were 571 and 576 nm, respectively. This red-shift in the LSPR band of Au@PL indicates the aggregation of Au@PLs, due to the changes in dielectric constant at the bare surface of AuNP [15]. According to our previous study for the AuNPs functionalized with zwitterionic lipids of DMPC (Au@DMPC), the LSPR band red-shifted to 580 nm. After sonication, the LSPR band blue-shifted to 573 nm. Therefore, the red-shift of the LSPR peaks represents the degree of assembly (or aggregation) of Au@PLs. The assembled degree of Au@PL in this study was smaller as compared to neutral Au@DMPC (LSPR: 580 nm), due to the electrostatic repulsion by anionic phospholipids, DMPS.

**Figure 2:**
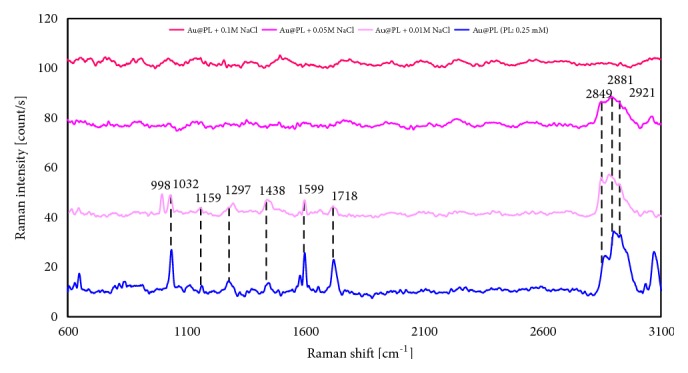
Raman spectra and of Au@PL with addition of NaCl in different concentration.

The effect of NaCl to Au@PL was observed by UV-vis (Figure 1) and Raman spectroscopy (Figure 2). The LSPR band of AuNPs blue-shifted from 576 nm to 562 nm, the profile was broadened, and the intensity decreased. The LSPR band of AuNPs was sensitive to their environment, shapes, and sizes. The addition of NaCl will induce the aggregation of AuNPs with red-shift and broad LSPR band [11, 16]. Herein, the LSPR band of Au@PL was broad but blue-shifted when the concentration of NaCl exceeded 0.05 M which indicated that NaCl induced Au@PL rather dispersed, instead of aggregation. As shown in Figure 2, the Raman intensity of Au@PL decreased as the amount of NaCl increased that showed the same tendency as UV-vis spectra. Therefore, the SERS properties are strongly related to the plasmon resonance of AuNPs. Moreover, the peaks of COOH and NH_3_^+^ disappeared which represented the loss of H-bond among DMPS. It is considered that NaCl shielded the surface of Au@PL, and disturbed the H-bond network so that originally aggregated AuNPs were forced to be away from one another. Nevertheless, without H-bond, the increasing amount of NaCl made Au@PL unstable and resulted in precipitation. On the other hand, C-H stretching around 2800-3000 cm^−1^ shifted to lower frequency and was similar to Au@DMPC [11]. Consequently, it is supposed that NaCl destroyed H-bond between DMPSs that enlarged the distance between Au@PLs, resulting in the decreased Raman enhancement.

### 3.2. Aggregation Behaviors of A*β* in the Presence of PL Vesicle and Au@PL

**Figure 3:**
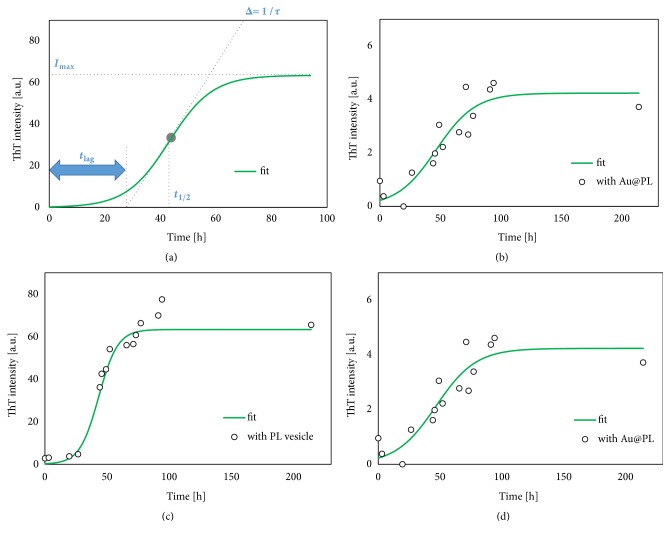
Time course of ThT fluorescence during A*β* fibrillation, and sigmoidal fitting for kinetics. (a) outline, (b) control, (c) with PL vesicle, and (d) with Au@PL.

The kinetic of amyloid fibrillation was characterized by ThT fluorescence method [8]. The results were analyzed based on a sigmoidal curve fitting (Figure 3(a)) (eq. (1)). The fitting curves were shown in Figures 3(b)–3(d) and then the obtained kinetic parameters are summarized in Table 1. A*β* fibrillation process can be explained as in the following three phases: (1) a lag phase where A*β* starts to change conformation and to form oligomers, (2) an elongation phase where A*β* begins to fibrillation that drastically increases the ThT fluorescence, and (3) an equilibrium phase where the fibrillation process is completed. The times reaching to 50% fibrillation (t_1/2_) were almost same, while the lag time until starting A*β* fibrillation (t_lag_) was fasten in the presence of PL vesicles. Then, the total time for maturation of fibril maturation (t_1/2_-t_lag_) became greater in the presence of PL vesicles, indicating that the negative charged PL membrane makes the fibrillation of A*β* slower. However, the overall fibrillation was promoted by PL vesicles, because the *I*_max_ became higher. In previous studies, anionic PL vesicles could accelerate adoption of antiparallel *β*-sheet structure with the formation of large fibril aggregation which is the toxicity of A*β* toward PL [8, 9]. A*β* attracted by head group of anionic PL so the local concentration of A*β* increases. Based on the membrane-enabled adoption, A*β* tends to aggregate into *β*-sheet [17–19]. Additionally, some part of A*β* will insert in membrane as *α*-helix or random coil with hydrophobic interaction [20]. In this study, the anionic PL accelerated the A*β* fibrillation, which is consistent with previous studies.

In the presence of Au@PL, the total fibril amount was drastically decreased. Theoretically, under the same membrane composition of PLs, it is then expected that the interaction of PL molecules and A*β* should be the same. Although the PL membranes could potentially promote fibrillation, the PL membrane constructed on AuNP inhibited A*β* fibrillation, indicating that the A*β* interaction toward PL and Au@PL is proceeded in a different mechanism. It has been proposed that aggregation prone site of A*β* is consisted with hydrophobic and anionic amino acids so that its fibrillation tends to be inhibited by binding to hydrophobic or positively charged surface [9, 17, 18]. This suggests that the aggregation prone site of A*β* is blocked by interior layer of Au@PL.

### 3.3. MSERS of Au@PL and A*β*

**Figure 4:**
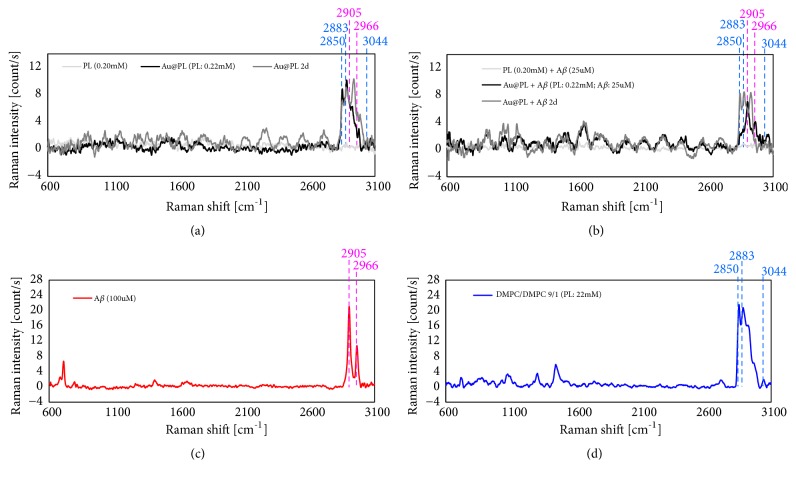
Raman spectra of (a) Au@PL and (b) Au@PL with A*β* 25 *μ*M before (gray line) /after (black line) 2 days incubation at 37°C. The corresponding Raman spectra of (c) A*β* and (d) PL vesicles are shown for comparison.

Au@PL with/without addition of A*β* was measured by Raman spectroscopy before and after incubation in 37°C (Figures 4(a) and 4(b)). The spectra of pure A*β* and PL vesicle are shown in Figures 4(c) and 4(d), respectively. In addition, the enhancement factor (EF) of C-H stretching of PL was calculated by eq. (3). The EF values of 2850 cm^−1^ at 0-hour and 2-days incubation were 20.5 and 38.4, suggesting the interaction between A*β* and Au@PL during incubation. Comparing Raman spectra of Au@PL and Au@PL with addition of A*β* shown in Figures 4(a) and 4(b), Raman signals at 1031 cm^−1^, 1374 cm^−1^, 2905 cm^−1^ and 2966 cm^−1^ appeared in the presence of A*β* before incubation. Based on studies of Hu et al. and Raman spectra of pure A*β* (Figure 4(c)), these four peaks are attributed to A*β* [20]. It is suggested that A*β* probably appeared near hot spot of nanoparticles so its Raman was enhanced. Moreover, the COOH stretching at 1721 cm^−1^ became weaker in the presence of A*β* (Figures 4(a) and 4(b), gray line). In previous research, A*β* tends to be attracted by PL via electrostatic interaction more than hydrophobic interaction [17]. As a result, we assume that A*β* is attracted by DMPS via electrostatic interaction so that the intermolecular H-bonding of DMPS might be partly destroyed.

After 2 days of incubation, interaction between Au@PL and A*β* achieved to equilibrium. The four peaks mentioned above still existed. Another Raman signals at 992 cm^−1^ and 1435 cm^−1^ appeared. These observed signals indicate A*β* is right in the hot spot region and the Phe and CH functional group are reactive parts of A*β* with PL. It is noted that Phe is part of the aggregation prone site of A*β*. Therefore, its fibrillation might be influenced. Additionally, COOH stretching at 1721 cm^−1^ disappeared but COO^−^ and NH_3_^+^ signals still remained. It is assumed that A*β* interferes with intermolecular H-bonding via reacting with COO^−^ and NH_3_^+^. The presence of *β*-sheet structure will show a shift of 1374 cm^−1^ to 1381 cm^−1^ and its amid III will appear at 1240 cm^−1^ for *β*-sheet and 1260 cm^−1^ for *α*-helix [20]. Moreover, the aggregation of A*β* will find increased intensity of Phe and Tyr at 1003 cm^−1^ and 1188 cm^−1^, respectively. However, there is only increased intensity of Phe without any appearance of *β*-sheet or *α*-helix could be observed from our results. The absence of secondary structure indicates A*β* probably appeared in random coil. This result agrees with the results from ThT fluorescence that Au@PL inhibits the fibrillation of A*β*.

As A*β* inserts in membrane, its structure is restricted and difficult to form *β*-sheet [17]. The unobserved *β*-sheet signals in Raman and ThT fluorescence are corresponding to this argument. It has been reported that inhibition of fibrillation is mainly dependent on blocking aggregation prone sites of A*β* followed by the conformational change of A*β* becoming unable to aggregate [7]. In this case, negatively charged Au@PL has little opportunity to bind to the aggregation prone site since it is consisted of hydrophobic and negatively charged residues [4–6].

**Figure 5:**
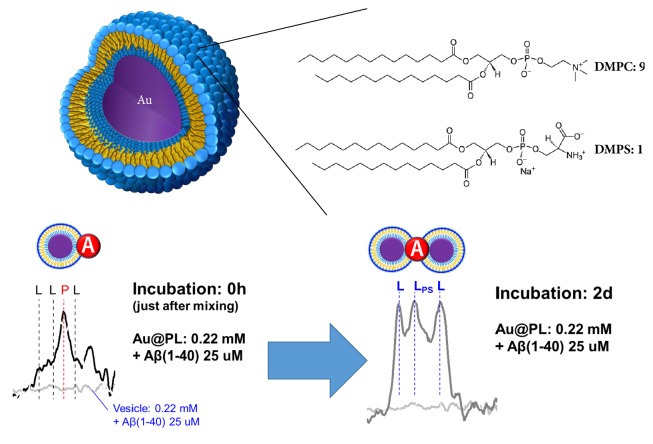
Summary of this study.

The addition of A*β* like addition of NaCl might induce aggregation of AuNPs whose kinetics is much faster than that of fibrillation. The EF values were increased in the presence of A*β* after incubation: EF = 8.8 without A*β*, EF = 20.5 with A*β*. Therefore, the aggregation of Au@PLs induced by A*β* is not only because of the change of ionic strength of surroundings but also strong interaction toward PL (Figure 5). It is noted that the aggregation prone site of Phe appeared in hot spots of Au@PL which make certain that A*β* is inside of aggregated Au@PLs. As a result, we suggested that binding of A*β* on Au@PL through COO^−^ and NH_3_^+^ of DMPS resists H-bonding [21–23]. Instead of pushing Au@PL away, the attraction of A*β* toward DMPS pulls them closer so that Au@PL aggregates. At the same time, A*β* attracted by hydrophobic part of PL and inserts into lipid membrane which makes distance among Au@PL gets shorter increasing Raman enhancement. Finally, the aggregated Au@PL blocks A*β* monomers to inhibit further fibrillation.

## 4. Conclusions

In conclusion, the presence of DMPS in PL stabilizes Au@PL with intermolecular H-bonding. The addition of NaCl will cover the surface of Au@PL and resist the H-bonding network resulting in precipitation of Au@PL. When A*β* is added to Au@PL solution, it behaves like NaCl to obstruct H-bond network by electrostatic attraction with COO^−^ and NH_3_^+^. Rather than keeping Au@PL away from each other like the influence of NaCl, the attraction of A*β* toward PL pull Au@PL together. Moreover, the insertion of A*β* in PL makes distance among Au@PL even closer than untreated Au@PL results in the highest EF values among all samples. However, the aggregated Au@PL with A*β* makes it difficult to fibrillation which is different from the incubation of PL with A*β*. Although, the result of fibrillation is different, the reactive site of PL with A*β* might be the same for the kinetics of deformation and elongation of A*β* have the same tendency. Therefore, the impact of A*β* with lipid membrane has been further understood via high-sensitive MSERS technique.

## Figures and Tables

**Table 1:** Kinetic parameters in A*β* fibrillation.

	t_1/2_ [h]	t_lag_ [h]	t_1/2_-t_lag_ [h]	*I*_max_ [a.u.]
Control	45.5 ± 3.4	37.9 ± 5.5	7.7 ± 5.4	49.5 ± 6.3

PL vesicle	57.2 ± 17.9	41.3 ± 25.7	16.3 ± 10.1	60.7 ± 17.9

Au@PL	58.2 ± 16.5	30.0 ± 22.6	27.5 ± 6.4	17.2

## Data Availability

The data used to support the findings of this study are included within the article.

## References

[1] Sasahara K., Morigaki K., Shinya K. (2013). Effects of membrane interaction and aggregation of amyloid *β*-peptide on lipid mobility and membrane domain structure. *Physical Chemistry Chemical Physics*.

[2] Arosio P., Knowles T. P. J., Linse S. (2015). On the lag phase in amyloid fibril formation. *Physical Chemistry Chemical Physics*.

[3] Nielsen L., Khurana R., Coats A. (2001). Effect of environmental factors on the kinetics of insulin fibril formation: Elucidation of the molecular mechanism. *Biochemistry*.

[4] Mirsadeghi S., Dinarvand R., Ghahremani M. H. (2015). Protein corona composition of gold nanoparticles/nanorods affects amyloid beta fibrillation process. *Nanoscale*.

[5] Cabaleiro-Lago C., Quinlan-Pluck F., Lynch I. (2008). Inhibition of amyloid *β* protein fibrillation by polymeric nanoparticles. *Journal of the American Chemical Society*.

[6] Kim Y., Park J., Lee H., Nam J. (2016). How Do the Size, Charge and Shape of Nanoparticles Affect Amyloid *β* Aggregation on Brain Lipid Bilayer?. *Scientific Reports*.

[7] Mahmoudi M., Kalhor H. R., Laurent S., Lynch I. (2013). Protein fibrillation and nanoparticle interactions: Opportunities and challenges. *Nanoscale*.

[8] Shimanouchi T., Shimauchi N., Ohnishi R. (2012). Formation of spherulitic amyloid *β* aggregate by anionic liposomes. *Biochemical and Biophysical Research Communications*.

[9] Kumar S., Rezaei-Ghaleh N., Terwel D. (2011). Extracellular Phosphorylation of the Amyloid *β*-peptide Promotes Formation of Toxic Aggregates during the Pathogenesis of Alzheimer’s Disease. *The EMBO Journal*.

[10] Altuntas S., Buyukserin F. (2018). Fabrication of thioflavin-T-modified nanopillared SERS substrates for ultrasensitive beta-amyloid peptide detection. *Journal of Raman Spectroscopy*.

[11] Suga K., Yoshida T., Ishii H. (2015). Membrane surface-enhanced Raman spectroscopy for sensitive detection of molecular behavior of lipid assemblies. *Analytical Chemistry*.

[12] MacDonald R. C., MacDonald R. I., Menco B. P. M., Takeshita K., Subbarao N. K., Hu L.-R. (1991). Small-volume extrusion apparatus for preparation of large, unilamellar vesicles. *Biochimica et Biophysica Acta (BBA) - Biomembranes*.

[13] Takayama M., Itoh S., Nagasaki T., Tanimizu I. (1977). A new enzymatic method for determination of serum choline-containing phospholipids. *Clinica Chimica Acta*.

[14] Batenjany M. M., Wang Z.-Q., Huang C.-H., Levin I. W. (1994). Bilayer packing characteristics of mixed chain phospholipid derivatives: Raman spectroscopic and differential scanning calorimetric studies of 1-stearoyl-2-capryl-sn-glycero-3-phosphocholine (C(18): C(10)PC) and 1-stearoyl-2-capryl-sn-glycero-3-phospho-N-trimethylpropanolamine (C(18): C(10)TMPC). *Biochimica et Biophysica Acta (BBA) - Biomembrane*.

[15] Kočišová E., Procházka M. (2011). Drop‐coating deposition Raman spectroscopy of liposomes. *Journal of Raman Spectroscopy*.

[16] Wang G., Sun W. (2006). Optical limiting of Gold nanoparticle aggregates induced by electrolytes. *The Journal of Physical Chemistry B*.

[17] Rai D. K., Sharma V. K., Anunciado D. (2016). Neutron Scattering Studies of the Interplay of Amyloid Beta-Peptide (1−40) and an Anionic Lipid 1,2- Dimyristoyl-sn-Glycero-3-Phosphoglycerol. *Scientific Reports*.

[18] Anunciado D., Rai D. K., Qian S., Urban V., O'Neill H. (2015). Small-angle neutron scattering reveals the assembly of alpha-synuclein in lipid membranes. *Biochimica et Biophysica Acta*.

[19] Lee C.-C., Sun Y., Huang H. W. (2010). Membrane-mediated peptide conformation change from *α*-monomers to *β*-aggregates. *Biophysical Journal*.

[20] Hu Z., Wang X., Wang W., Zhang Z., Gao H., Mao Y. (2015). Raman spectroscopy for detecting supported planar lipid bilayers composed of ganglioside-GM1/ sphingomyelin/cholesterol in the presence of amyloid-*β*. *Physical Chemistry Chemical Physics*.

[21] Jarmelo S., Carey P. R., Fausto R. (2007). The Raman spectra of serine and 3,3-dideutero-serine in aqueous solution. *Vibrational Spectroscopy*.

[22] Jarmelo S., Reva I., Carey P. R., Fausto R. (2007). Infrared and Raman spectroscopic characterization of the hydrogen-bonding network in l-serine crystal. *Vibrational Spectroscopy*.

[23] Socrates G. *Infrared and Raman Characteristic Group Frequencies: Table and Charts*.

